# Quantifying the impact of health service delivery barriers on access to healthcare: a case study of antiretroviral therapy in Mali

**DOI:** 10.1136/bmjgh-2025-019476

**Published:** 2026-06-19

**Authors:** Pablo Timoner, Fleur Hierink, Nicolas Ray, Ousmane Toure, Hamsatou Cissé, Ousmane Sy, Caroline Fuhrer, Samuel Petragallo

**Affiliations:** 1 GeoHealth Group, Institute of Global Health, Faculty of Medicine, University of Geneva, Geneva, Switzerland; 2 Institute of Environmental Sciences, University of Geneva, Geneva, Switzerland; 3 Health Resources and Services Availability Monitoring System (HeRAMS) Initiative, World Health Organization, Geneva, Switzerland; 4 Health Resources and Services Availability Monitoring System (HeRAMS), World Health Organization, Bamako, Mali; 5 Unité de Mise en Oeuvre du Renforcement du Système de Santé, Gouvernement du Mali Ministère de la Santé et du Développement Social, Bamako, Mali; 6 Direction Générale de la Santé et de l’hygiène Publique, Gouvernement du Mali Ministère de la Santé et du Développement Social, Bamako, Mali

**Keywords:** Delivery of Health Care, Geographic information systems, Health services research, Health Services Accessibility, Africa South of the Sahara

## Abstract

Achieving universal health coverage is a key component of the Sustainable Development Goals, focusing on equitable access to quality health services and minimising financial hardship. While strategies often target the demand-side, supply-side barriers such as travel time and facility-level constraints are often overlooked. Accurately quantifying who is affected by these barriers and identifying their locations and specific barriers is critical to improving service delivery. This study examines these barriers in Mali, a country with significant health system challenges exacerbated by high fertility rates and political instability. Using the WHO’s Health Resources and Services Availability Monitoring System, we conduct an analysis of the geographic accessibility of antiretroviral therapy (ART) services. Our aim is to estimate the number of people affected by supply-side barriers by using a geographic accessibility model that calculates travel time to facilities with ART services. The analysis applies a least-cost path algorithm to assess accessibility, where ART services are defined as accessible within 2 hours travel time. People within this range with available services have access while those outside are geographically constrained. For those within 2 hours but without ART access, we identified and quantified facility-specific barriers. The results show that nearly 2.7 million Malians do not have timely access to ART within 2 hours. For about 70%, distance is the main barrier, while the rest face facility-level issues such as the fact that the service is not being planned in the facility, lack of medical supplies and lack of training. This study offers important insights for targeted interventions to scale up ART provision and provides a scalable model for other health services and contexts.

**WHAT IS ALREADY KNOWN ON THIS TOPIC**

- In low- and middle-income countries (LMICs), healthcare access is often hindered by both demand-side and supply-side barriers, such as geographic inaccessibility and inadequate service availability. These challenges lead to preventable deaths, with 8.6 million deaths in 2016 potentially having been preventable through improved healthcare access and quality of care.

**WHAT THIS STUDY ADDS**

- This study uniquely examines both travel time and facility-based barriers to antiretroviral therapy (ART) in Mali, finding that nearly 2.7 million people lack ART access. About 70% face geographical and distance issues, while others are hindered by unplanned services, supply shortages and lack of training.

**HOW THIS STUDY MIGHT AFFECT RESEARCH, PRACTICE OR POLICY**

- Our analysis guides targeted interventions to improve ART service delivery by highlighting supply-side barriers with HeRAMS (Health Resources and Services Availability Monitoring System) data. By identifying geographi variations in service availability and accessibility, this approach supports the design of context-specific strategies to strengthen ART delivery. Our findings underscore the need for regionally tailored health system policies that address local service gaps and access constraints.

## Introduction

 Access to healthcare is essential for achieving universal health coverage (UHC) by 2030, as outlined in target 3.8 of the Sustainable Development Goals.^1^ UHC mainly encompasses two fundamental commitments: (1) ensuring equitable access to quality health services and (2) preventing financial hardship for individuals receiving healthcare.^2 3^

While ensuring access to healthcare involves various critical components (ie, availability, accommodation, geographic accessibility, affordability and acceptability),^4^,^6^ efforts often concentrate on addressing demand-side barriers.^3 7^ Strategies to reduce demand-side barriers target factors influencing the utilisation of health services at the individual, household or community level. These include increasing (domestic) health financing to lower out-of-pocket expenses and raising awareness to promote health-seeking behaviour.^8^ However, supply-side barriers, such as the availability of health services, are often underrepresented in accessibility assessments. These barriers can be categorised into two dimensions. The first dimension is travel time (or distance), which refers to the lack of facilities offering the service within a reasonable travel time (or distance) from the populations of interest. The second dimension, referred to as facility-level barriers, involves barriers such as shortages of medical staff and insufficient medical equipment that prevent facilities that are within an acceptable distance from delivering the intended service. Travel time and facility-level barriers can significantly hinder progress towards UHC and have a notable impact on the quality of care and health outcomes. It is crucial to quantify the number of individuals affected by barriers and to pinpoint where and what specific barriers are hindering quality service delivery.

The Health Resources and Services Availability Monitoring System (HeRAMS) by the WHO plays a vital role in monitoring essential health resources and services.^9 10^ HeRAMS enables the monitoring of the availability of individual services at service delivery points, such as health facilities. Through its geospatial modelling service, HeRAMS is able to model travel time and distance (ie, covers the first part of the barrier analysis). When a service is expected to be available but cannot be provided, or is not delivered at the required standard, HeRAMS helps systematically analyse the main barriers at the facility level that hinder effective service delivery. By combining data on travel time with these facility-level barriers, HeRAMS offers a comprehensive view of the supply-side obstacles that affect healthcare delivery.

One of the countries where HeRAMS has been deployed is Mali. The health system in Mali faces major challenges such as the inadequate, unstable and uneven distribution of qualified human resources, compromising its ability to guarantee a minimum quality of care and meet the needs of a growing and urbanising population.^11^ This situation is driven by Mali’s high fertility rate, which at 5.4 is the sixth highest in the world in 2025 and has only decreased by 1.4 since 1960, according to the United Nations Population Fund.^12^ Since 2012, Mali has been going through a political and security crisis that has worsened an already concerning health situation.^11^

In this context, it is crucial to quantify and understand the relative importance of the various barriers that hinder the availability of essential health services. This is particularly important as in 2019 Mali announced a major health system reform that required about US$120 million in additional funding to provide free health services to children under 5 and people over 70.^13^ While the rollout is underway, ongoing challenges—including the COVID-19 pandemic and political instability—have led to persistent funding gaps, even for resources initially granted by the Global Fund and Gavi.^14^ The main concern with this reform is that pooling external funds and injecting them into the health system will not achieve the desired results on the supply side if health facilities are not adequately staffed, equipped with medicines, security and clean water.^13^

This concern is relevant as previous research by Touré^15^ has shown that the removal of user fees does not translate to improvements in quality of care. In Mali, user fee exemption policies exist for certain health services, such as HIV/AIDS treatment, caesarean sections and malaria treatment, which aim to eliminate financial barriers on the demand side. Since 2006, Malian law has mandated the removal of user fees for all individuals living with HIV/AIDS, covering access to antiretroviral therapy (ART), contraceptives for both men and women, consultation costs, analyses and screenings, as well as biological monitoring and treatment of opportunistic diseases.^15^ The exemption for ART has received significant support from international partners. Currently, approximately 18.3% (ie, US$6.1 million) of all expenditures on HIV/AIDS, and 13% (or US$1 million) of spending on HIV treatment interventions, are funded domestically.^16^ International funding still accounted for a substantial 82% of Mali’s HIV/AIDS expenditures.^16^ The reliance on international donor support for HIV/AIDS management in Mali has led to heavy dependence on external funding and the development of a two-tiered system, where the public sector misses out on the benefits provided to the associative sector, ultimately hindering broader health system integration and expansion while failing to address underlying supply-side barriers. Several disruptions in the provision of quality HIV/AIDS care have been observed during the last decade, including periods of insufficient skilled staff, faulty equipment and long waiting times.^15^

To effectively address health service delivery barriers and formulate adequate policies, it is essential to identify and quantify the impact of travel time as well as facility-level barriers on healthcare access. In this paper, we aim to estimate the number of people impacted by supply-side health service delivery barriers. We use Mali and health services for HIV as a case study, given the importance of adherence to ART and the continuity of care over the long course of HIV infection.

In Mali, HeRAMS currently provides regular information on 2676 health facilities including barriers that hinder ART service delivery.^17^ To estimate the number of people impacted by health service delivery barriers, we employ a geographic accessibility model that calculates travel time to health facilities where ART services are available or not available. We define physically accessible ART services as those within a 2-hour travel time. Individuals living within this threshold, where services are available, are considered to have physical access to ART. Those living beyond this threshold lack access due to geographical constraints. For populations within 2 hours of a facility where ART services are not available, we quantify the impacted population by identifying the nearest facility for each person, assessing its service delivery barriers and summarising the total number of people affected by each barrier. We quantify access to ART services using a 2-hour travel time threshold. The 1-hour and 2-hour thresholds are both rooted in emergency care contexts, the former reflecting the ‘golden hour’ for life-saving interventions^18^ and the latter commonly used for emergency obstetric care.^19 20^ However, because ART access concerns continuity of care rather than acute medical response, the 2-hour benchmark provides a more realistic and appropriate measure of geographic accessibility. While the focus of this article is on ART services in Mali, this approach is scalable to other health services and contexts.

## Methods

### Data

Health facility data for Mali were obtained from the HeRAMS database as of 6 July 2024. For the purpose of this study, we focused only on public health facilities that form the core of Mali’s three-tiered pyramidal healthcare system. These included community health centres (CHCs), reference health centres (RHCs) and hospitals (Hs). In total, there were data for 1579 facilities. CHCs accounted for the vast majority at 95% (n=1502) of observations, while RHCs made up 4% (n=63) and Hs only 1% (n=14) of the public health facilities. Data included the location of health facilities, the availability of ART for HIV at the facility level, and when not available, the barriers to its delivery. In HeRAMS, the health service can be ‘available’, ‘partially available’ or ‘not available’ when considered to be ‘normally provided’ or ‘not normally provided’ (online supplemental figure 1). To simplify this study, we considered the service to be available even when it was indicated as partially available. When ‘not available’, the obstacles to its delivery, which are not mutually exclusive, can be ‘lack of financial resources’, ‘lack of medical equipment (incl. logistic, fuel, vehicles, maintenance)’, ‘lack of medical supplies (drugs and consumables)’, ‘lack of staff’ or ‘lack of training’. For geographic accessibility modelling and the service coverage assessment, vector data of administrative boundaries, the road network, rivers, gridded data of land cover, digital elevation model and spatial distribution of the population, and information regarding local transportation modes and travel speeds were also required (online supplemental table 1).

High-resolution census-based gridded population density estimates of the target population (ie, the general population), constrained to the building footprint, were sourced from WorldPop in collaboration with the Institut National de la Statistique du Mali and corrected with 2022 population data from the National Office of Reproductive Health (ONASR) (data by ADM1).^21^ The values were further corrected to ensure that the regional population corresponds to the regional census data for 2022 provided by the ONASR. The vector data of administrative boundaries, roads and watercourses were obtained from the Geographic Institute of Mali. The land cover and elevation data were sourced from the European Union’s Copernicus programme^22^ and the international initiative Shuttle Radar Topography Mission,^23^ respectively. Finally, as part of the national prioritisation workshop for emergency obstetric and newborn care (EmONC) organised from 6 June 2022 to 9 June 2022, and led by the Ministry of Health and Social Development, national experts defined the motorised travel scenario (ie, the modes and speeds capturing the health seeking behaviour of the target population when using motorised transport) following the standard operating procedure proposed by Molenaar *et al*^24^ and by subdividing the country into three eco-geographical zones with the following grouping of Regions:

Scenario 1: Kayes, Koulikoro, Ségou, Bamako, Sikasso. The bulk of the paved and improved roads are concentrated in the southern part of the country, although they are not dense there and many are in bad condition.

Scenario 2: Mopti. This region is characterised by a high hydrographic density with highly developed river transport.

Scenario 3: Tombouctou, Gao, Menaka, Kidal, Taoudénit. These regions are characterised by their low density of the road network. River transport is also developed there except in Menaka.

Because travel modes and speeds vary considerably between subnational regions, each region was assigned its own travel scenario. The friction surface was therefore developed using localised health-seeking behaviour patterns and corresponding cost values applied within the least-cost path algorithm in AccessMod. This approach allowed travel impedance to reflect regional transport conditions and accessibility differences rather than relying on a single national assumption.

Colleagues from the WHO country office verified that the motorised travel speeds accurately reflected the identified travel scenarios and corresponded to the transport modes and speeds commonly used by the general population.

Walking speeds, applied to landcover types and terrains where motorised transport was not feasible, were adopted from Watmough *et al* and ranged from 1.5 km/hour to 3 km/hour depending on terrain type.^25^ Details of the travel scenarios are provided in online supplemental table 2.

### Analysis

To model geographic accessibility, we used the open-source software AccessMod,^26^ a tool developed by the University of Geneva in collaboration with the WHO. It allows the modelling of the population’s physical accessibility to health facilities based on different modes of transportation and travel speeds within a given geographic region. The modelling of physical accessibility is based on travel time rather than distance. This approach provides a more realistic representation by taking into account movement facilitators such as road networks and means of transportation, travel speeds on those networks, as well as movement constraints like topography, lakes, rivers, and agricultural areas. Additionally, AccessMod enables the modelling of movements outside the road network, which is particularly useful in regions where travel does not solely occur on roads or where data on road networks were incomplete.

We created the travel impedance surface at the 90 m resolution of the WorldPop population raster so that the population count, our main denominator, only required reprojection to UTM 30N (EPSG:32630) and no further resampling, reducing the risk of value distortion. The landcover and digital elevation model (DEM) were reprojected, resampled, and aligned to the population raster, using nearest neighbour for landcover and bilinear interpolation for the DEM. All spatial layers were imported into AccessMod and merged into a single landcover layer, with some waterbodies treated as complete barriers unless crossed by roads, while others were considered navigable by motorised boats. All the spatial preprocessing was done in R^27^ using the ‘inAccessMod’ package.^28^

**Figure 1:**
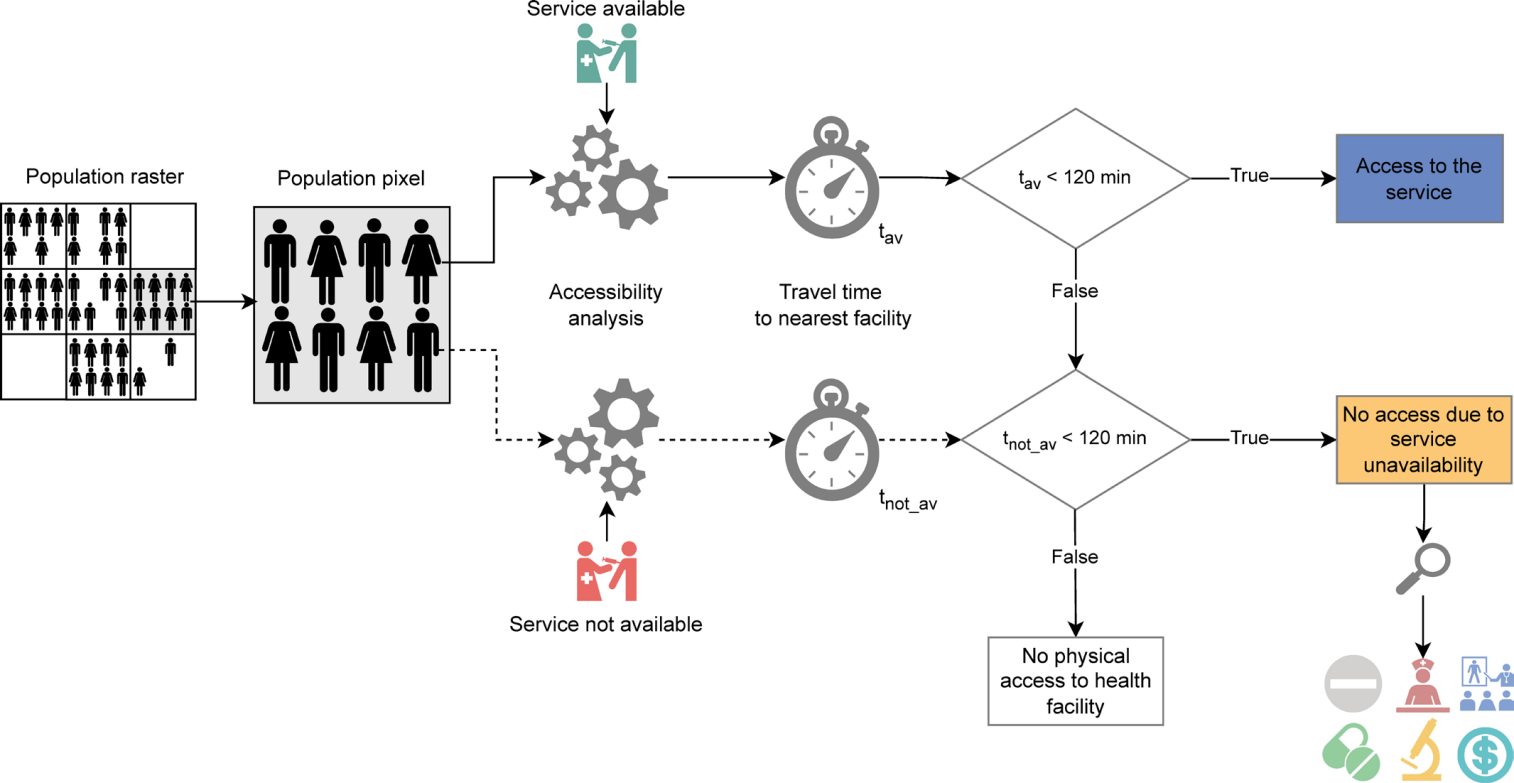
A flow chart summarising the dual-model methodology. The analysis uses a first model mapping travel times to facilities where the service is available, and a second for where the service is unavailable. The diagram illustrates the decision pathway for each pixel, determining the accessibility based on a 120-minute travel time threshold. When the nearest available service exceeds this limit, the travel time to the nearest facility lacking the service is assessed. Population with travel times to any health facility over 120 min are considered to have no physical access. Furthermore, for population with access only to facilities lacking the service, further analysis categorises the causes of service unavailability based on HeRAMS information. HeRAMS, Health Resources and Services Availability Monitoring System.

A dual-model methodology was used. A first model considered only health facilities where the service was available, and a second model considered only health facilities where the service was not available due to specific barriers at the supply-side (eg, human resources, financial constraints) (figure 1). Therefore, we calculated two travel times for each populated pixel in the country (hereafter referred to as population pixels): (1) travel time to the nearest health facility offering the service and (2) travel time to the nearest facility where the service was unavailable. When the nearest health facility providing the service was not accessible within 120 min, we evaluated the travel time to the nearest facility that did not provide the service. If it exceeded 120 min, the population was considered to have no physical access to the service. Otherwise, we investigated the reasons for the service’s unavailability. These reasons may include the service not being planned (‘not normally provided’) or the presence of specific supply-side barriers to the service delivery, such as lack of training, medical supplies, equipment, staff or financial resources. When multiple barriers were mentioned, the affected population by each barrier corresponded to the total population divided by the number of barriers. While no guideline exists for geographic access to ART services, the 120 min threshold was chosen as a compromise between existing guidelines on accessing emergency care and existing literature indicating that people living with HIV/Aids are willing to travel further distances for ART.^29^ This analysis allowed us to quantify the absolute and relative impact of each cause (travel time, service not being planned, various barriers) on service accessibility, based on the number of affected individuals. It is important to note that the administrative boundaries of each region did not act as physical barriers, and the model assumed that the population could travel between regions to seek healthcare. While all statistics in the results are presented for the 120-minute travel time threshold and in some instances the 60-minute travel time threshold, we also generated outputs for thresholds of 90, 150 and 180 min.

### Uncertainty estimates

To account for any uncertainty around the assumed speeds of transport, we also considered two additional scenarios where speeds were either 20% slower or 20% faster than our proposed scenario. This approach to generating uncertainty estimate was adapted from Ouma *et al* and Hierink *et al*.^30 31^

### Patient and public involvement

It was not appropriate or possible to involve patients or the public in the design, or conduct, or reporting, or dissemination plans of our research.

## Results

The lack of medical supplies and the lack of training were the most frequently reported barriers to the delivery of ART in Mali’s CHCs that plan to provide this service, with 27% of the facilities being affected by at least one of these issues. Table 1 reflects the reported barriers preventing the service provision at the facility level.

**Table 1:** Relative and absolute numbers of the health facilities by type affected by the different barriers to the delivery of ART in Mali, including the number of health facilities in which the service is not planned.

	Community health centre	Reference health centre	Hospital
Lack of financial resources	1% (21)	–	–
Lack of medical equipment	5% (69)	–	–
Lack of medical supplies	21% (309)	5% (3)	–
Lack of staff	10% (149)	–	–
Lack of training	19% (282)	2% (1)	–
Not normally provided	24% (365)	8% (5)	36% (5)

ART, antiretroviral therapy.

**Figure 2:**
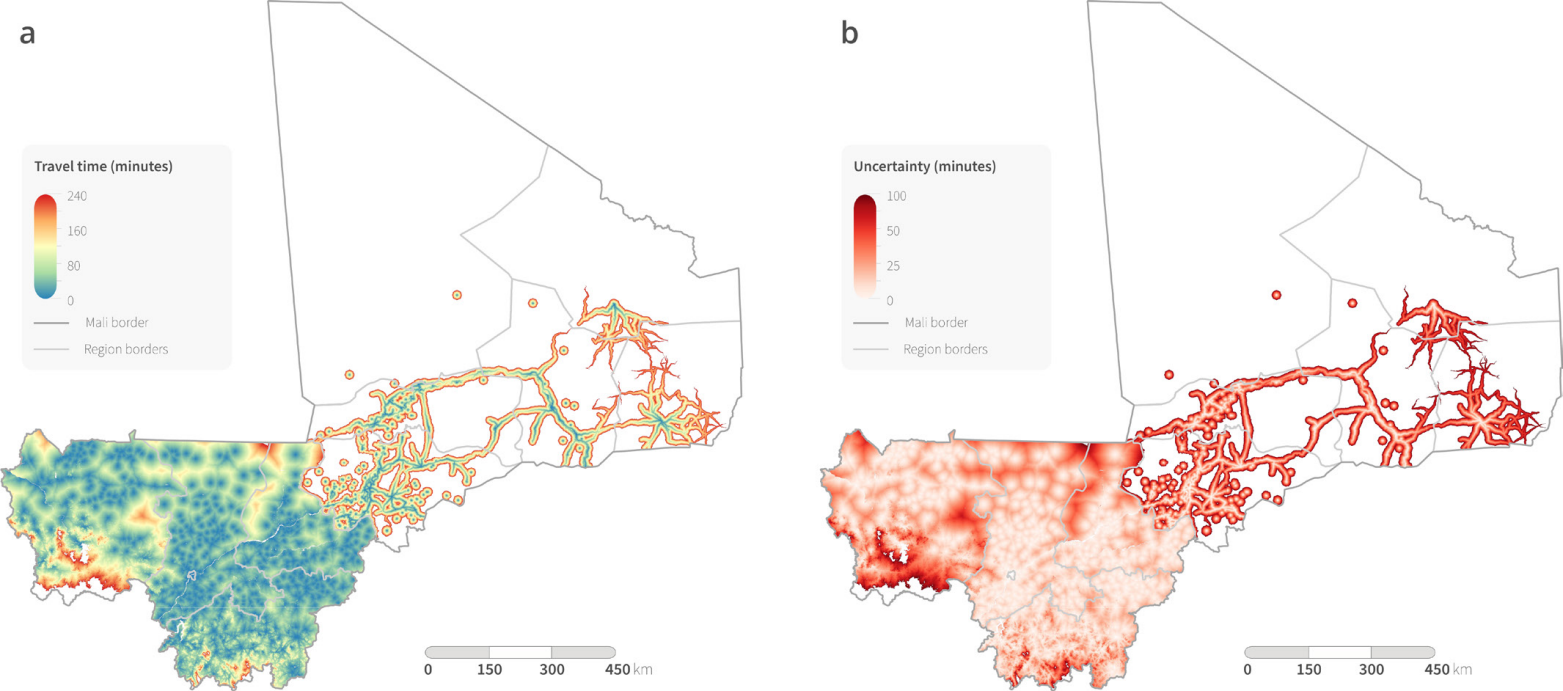
(**a**) Travel time map to health facilities providing antiretroviral therapy (ART) in Mali. Colours represent the travel time to the nearest facility up to 4 hours (240 min). (**b**) Uncertainty map, indicating the difference in travel time (in minutes) between the model considering −20% and +20% on the assumed travel speeds.

**Figure 3:**
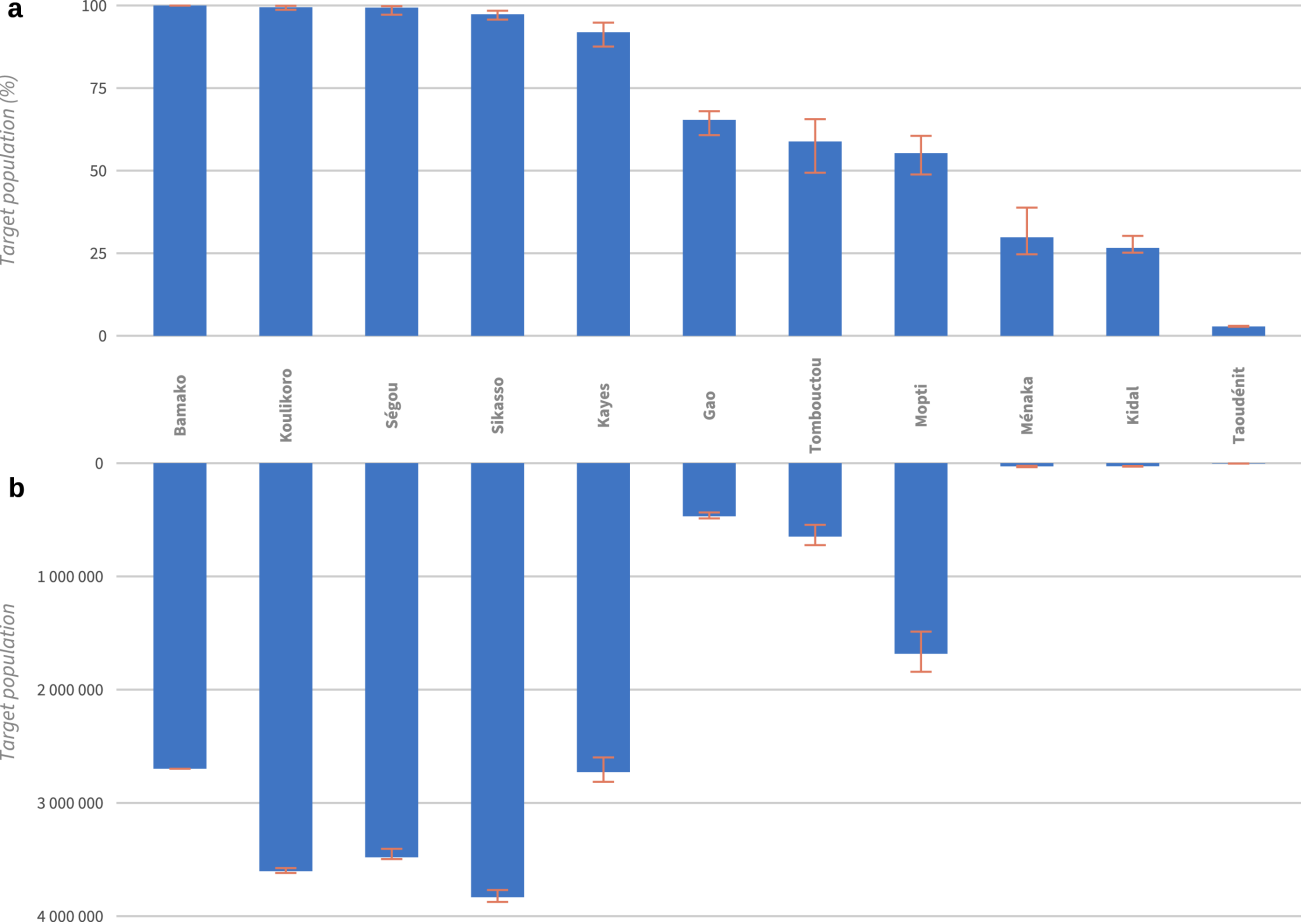
The accessibility coverage of ART within 120 min in (a) relative and (b) absolute values by region. The regions in the barplots are arranged in descending order of relative population coverage. Orange error bars indicate the coverage uncertainty, considering −20% and +20% on travel speeds. ART, antiretroviral therapy.

Figure 2a shows the travel time required to access a health facility providing ART in Mali and figure 2b shows the associated uncertainty around this estimate considering either 20% slower or faster speeds. The different behaviour of the model across regions can be explained, among other things, by considering different modes of transportation and speeds per region. Accessibility is highest in the more densely populated south-western regions, where 92%–99% of the population can reach ART services within 120 min, depending on the region. In contrast, coverage is lowest in Taoudénit (3%), Kidal (27%) and Ménaka (30%) (figure 3). Reducing the travel time threshold to 60 min results in a marked decline in accessibility, even in regions with comparatively better coverage. In Kayes, for example, accessibility falls from 91% to 74%, indicating that an additional hour of travel time enables roughly 500 000 more people to reach ART services. Only Bamako and Koulikoro maintain over 90% accessibility within 1 hour, while more than half of the other regions drop below 50%.

**Figure 4:**
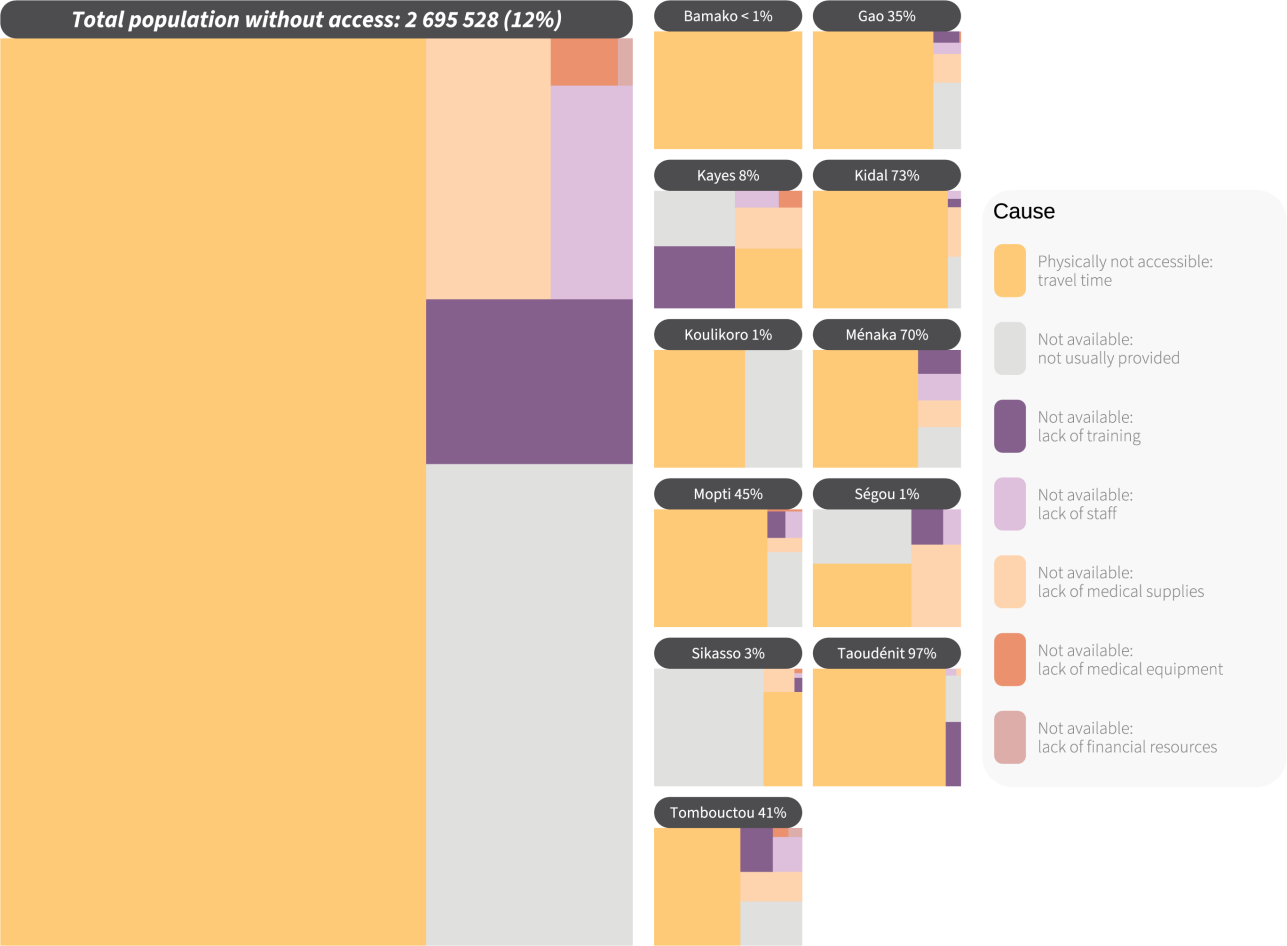
Treemap representing the causes of inaccessibility to the antiretroviral therapy (ART) in Mali for a maximum travel time of 120 min, according to the number of affected people. The percentages indicated for each region represent the percentage of the population without access to the service. The relative size of each cause of inaccessibility within a region is proportional to the percentage of the affected population.

At the national level, approximately 2.7 million people lack access to ART services within 120 min (2.3–3.3 million under ±20% travel speed scenarios), a number that rises to over 5 million when a 60-minute travel time threshold is applied (4.3–6.2 million under ±20% travel speed scenarios). In both cases, about 70% of this population live beyond the respective threshold to the nearest health facility, highlighting the role of geographic inaccessibility. The remaining 30% live within 2 hours of a facility but cannot access ART because nearby centres do not provide the service (figure 4). The fact that the service is not planned within the facilities, the lack of medical supplies and the lack of training represent the three most important obstacles to service delivery at the facility level. The impact of the different causes for inaccessibility varies widely across the regions. For instance, while travel time notably limits access to the service in Taoudénit and Kidal, it has little impact in Kayes, where access to the service is significantly affected by the lack of training and medical supplies. Overall, it is estimated that these two barriers potentially impact 200 000 people at the national level. The fact that the service is not planned in many health facilities further exacerbates the issue, affecting an additional 400 000 people.

## Discussion

Approximately 2.7 million people in Mali lack timely access to ART services. For about 70% of them, distance is the primary barrier. However, the remaining 30% encounter other, often overlooked obstacles. This means that nearly 1 million people, despite having physical access to health facilities, are hindered by facility-level barriers that impair service provision.

While assessing overall access to the health system is crucial, it only reveals part of the picture. Injecting (new) funding into the system will not achieve the desired impact unless it is allocated to address specific barriers—whether that means opening new health service delivery units in some areas or tackling medicine shortages and other obstacles in others. This is confirmed by previous research which has shown that, particularly in contexts where health systems rely primarily on external funding, policy reforms do not necessarily lead to sustainable integration or implementation of ART services in Mali.^15^ To better target where and which interventions and policy improvements are needed, it is necessary to monitor the availability of health services, the resulting gaps and related barriers. HeRAMS enables such monitoring in Mali.^17^

To illustrate this, the results of this case study indicate, among others, that the relatively low ART coverage in Kidal and Kayes should not be approached in the same manner. In Kidal, where travel time represents an important barrier, strategic placement of additional health centres or service delivery units (eg, community health workers) in optimally situated locations could help strengthen coverage. Conversely, coverage in Kayes, where lack of training is a main limitation, may be more effectively boosted through targeted training initiatives at existing facilities.

The impact of reduced access to ART on HIV/AIDS-related health outcomes in Mali is limited, however previous research in other sub-Saharan African countries shows that barriers to accessing ART care, including increased travel time and poor geographic accessibility, have been robustly associated with delayed or missed treatment initiation and reduced retention in care, which can be related to higher HIV-related morbidity and mortality and a greater burden of disease.^32^,^37^ For instance, Bilinski *et al*^33^ demonstrate in Malawi that geographic barriers contribute to substantial gaps in ART uptake and long-term health outcomes, while Siedner *et al*^37^ and Croome *et al*^34^ provide evidence from sub-Saharan Africa that greater distances to care facilities are linked to increased loss to follow-up. Similar patterns are to be expected for Mali.

While it is well-documented that the utilisation of HIV services is significantly influenced by distance, there is no established international guideline that specifies an optimal distance or travel time to facilities offering ART services. This stands in contrast to the clear guidelines for EmONC, which recommend that pregnant women should be able to reach an EmONC facility within 2 hours to ensure timely and life-saving care.^38^ Research underscores the impact of distance on ART initiation. For instance, a study conducted in rural South Africa demonstrated a 3% decrease in the odds of starting ART for every additional kilometre of distance from the nearest health clinic providing these services.^39^ Additionally, in Malawi, Palk *et al* identified a 1-hour travel time as optimal for maximising coverage of HIV therapy, indicating that shorter travel times could significantly enhance treatment access.^40^ However, other research suggests that people living with HIV/AIDS may be willing to travel further distances to access care, particularly when facing community stigma. For example, a study in South Africa^29^ has shown that individuals are sometimes prepared to travel more than 5 km to reach a clinic, to avoid local stigma and maintain privacy. In view of these findings, our decision to set a maximum travel time of 2 hours represents a balanced approach. It is consistent with the urgency of timely access to ART—similar to the 2 hour guideline for EmONC—while also recognising that people may be willing to travel longer distances to receive treatment.

Although our decision to adopt a 2-hour travel time threshold to present the results was carefully considered and based on the balance between ensuring timely ART access and accommodating the realities faced by people living with HIV/AIDS, we acknowledge that this threshold may still be perceived as somewhat arbitrary. Different travel time thresholds could indeed influence our findings and interpretations of the barriers encountered by individuals seeking ART services. For instance, if we had opted for a more restrictive threshold of 1 hour, as suggested by Palk *et al*^40^ for optimising HIV therapy coverage in Malawi, we would have observed a higher proportion of individuals without physical access to ART services—over 5 million people would lack access. This number would already drop to 1.8 million if we increased the travel time threshold to 3 hours. Such changes would, in turn, alter our assessment of the relative impact of various supply-side barriers, potentially highlighting different challenges in accessing care. To address this, we included additional travel time thresholds (ie, 60, 90, 150 and 180 min; see online supplemental figures 2–9) to analyse the number of people covered under these different cut-off values. However, the reasons for inaccessibility of ART services largely remain the same, with lack of physical access being the primary driver, followed by facilities not providing ART services, lack of medical supplies and insufficient training. While the percentages and absolute numbers vary between thresholds, the patterns remain consistent. This approach provides further insight into how varying thresholds affect physical access to timely ART, even though the precise reasons underlying these variations may not be fully captured.

Comparisons between our model and existing accessibility models could help assess the differences in results and provide insight into variation or uncertainty related to modelling assumptions. While we agree that such cross-model comparisons can be informative, we generally avoid relying on global or continental accessibility models to represent realistic access at national or subnational scales. For example, in Weiss *et al*,^41^ travel speeds are based on OSM data and broad assumptions on transport modes, combining global defaults, legal speed limits and generalised parameters where local data are lacking. This contrasts with our approach, which incorporates country-level and subnational consultations to define locally relevant travel modes and speeds. In addition, differences in landcover treatment and health facility datasets further limit direct comparability across studies.

Nevertheless, we visually compared travel time surfaces for Mali (online supplemental figure 10). The higher-resolution model (20 m) by Watmough *et al*^25^ reveals finer local accessibility patterns, while coarser resolutions (90 m in our study and 1 km in Weiss *et al*^41^) may overestimate accessibility under motorised travel assumptions. Despite these differences, all three models show similar broad patterns, with higher accessibility in the southwest and lower accessibility in the sparsely populated north, supporting the plausibility of our results.

Some limitations of this analysis must be acknowledged to appropriately interpret the results. Two key methodological constraints relate to our assumptions regarding facility capacity and potential patient bypassing behaviour. We assumed that a patient would go to the nearest health facility as long as the service is available, even though the theoretical number of potential patients captured by a health facility may exceed its capacity. Additionally, we did not account for the possible phenomenon of ‘by-passing’, whereby patients may not go to the nearest health facility for various reasons (eg, perceived or actual quality of care, financial barriers). Further studies could benefit from service usage data with household locations of patients to refine the true barriers and quantify the extent of the by-passing. One of the main sources of data uncertainty in our outcomes is the distribution and number of population estimates. Different gridded population datasets use varying methodologies, resulting in substantial differences in coverage estimates within geographic accessibility models. For example, Hierink *et al* reported that in Mali, 2-hour access to public health services ranged from about 30% to 70% in some regions depending on the dataset.^42^ In the absence of recent and reliable census data, it remains difficult to determine which gridded population product most accurately reflects reality. We therefore used WorldPop constrained estimates as the best available compromise between spatial resolution (~90 m for Mali), data availability and methodological suitability for accessibility modelling. Since geographic accessibility aims to represent population at place of residence to support effective microplanning and health system planning, constrained estimates—where population is allocated only to built-up areas—are more appropriate than unconstrained or ambient population datasets.

While we included a simple ±20% variation in travel speeds to illustrate the potential sensitivity of the results, this test should be viewed as indicative rather than exhaustive. A full uncertainty assessment—including variability in input data such as road networks, population density and travel mode transitions—was beyond the scope of this methodological study but represents an important direction for future work. Given the deterministic nature of the accessibility model and the lack of empirical uncertainty distributions for travel speeds, uncertainty was therefore explored through sensitivity analyses of travel speed parameters rather than through formal CIs.

In addition, while HeRAMS provides valuable insights into the functionality and availability of health services, several limitations should be considered when interpreting the findings. First, HeRAMS relies primarily on self-reported data from health facility focal points, which may introduce reporting bias or inaccuracies due to incomplete or outdated information. Additionally, data completeness and timeliness may vary across geographic areas and reporting periods, which can affect the representativeness and comparability of the results. To address these limitations, several strategies have been put in place. These include cross-validation of reported data with field assessments or other independent datasets, analysis of GPS coordinates accuracy and related corrections, regular training and capacity-building of HeRAMS focal points and enforcement of standardised reporting tools and harmonised data collection protocols to improve consistency across geographic areas.

This study employed a dual-model methodology to contextualise the accessibility barriers to ART across Mali’s diverse regions. A least-cost path algorithm was utilised to evaluate both the geographic accessibility of health facilities where the service was available and where the service was not. Concurrently, we conducted an in-depth analysis of HeRAMS data to identify the key supply-side barriers that impact population access to ART services. The analysis generated valuable insights to guide more tailored interventions seeking to expand coverage. However, opportunities remain to strengthen accessibility assessments. We encourage researchers to employ comprehensive methodologies that consider the full range of potential constraints influencing health service access in different countries, especially on the supply-side. A multidimensional perspective is needed to distinguish context-specific barriers requiring priority attention, and this was enabled here by leveraging the rich service-level data contained within HeRAMS information. This study is proof of concept, and our approach is scalable to other contexts and other health services informed in HeRAMS.

## Supplementary material

10.1136/bmjgh-2025-019476 online supplemental file 1

10.1136/bmjgh-2025-019476 online supplemental file 2

## Data Availability

Data are available on reasonable request.
