# Supplementary material for: Quantifying the impact of health service delivery barriers on access to healthcare: a case study of antiretroviral therapy in Mali
Source: BMJ Glob Health. 2026 Jun 19;11(6):e019476. doi: 10.1136/bmjgh-2025-019476 (PMC13289087; doi:10.1136/bmjgh-2025-019476)
Supplement: online supplemental file 1 [file bmjgh-11-6-s001.pdf]

BMJ Global Health Author Reflexivity Statement

Adapted from Morton, B., Vercueil, A., Masekela, R., Heinz, E., Reimer, L., Saleh, S., Kalinga, C., Seekles, M., Biccard, B., Chakaya, J., Abimbola, S., Obasi, A. and Oriyo, N. (2022), Consensus statement on measures to promote equitable authorship in the publication of research from international partnerships. Anaesthesia, 77: 264-276. https://doi.org/10.1111/anae.15597

**Study conceptualisation**	
1. How does this study address local research and policy priorities?	Ensuring equitable access to essential health services is central to achieving Universal Health Coverage (SDG target 3.8). In Mali, however, multiple supply-side barriers continue to limit the delivery of quality care. The health system faces an inadequate, unstable and uneven distribution of qualified human resources, one of the highest fertility rates globally, and ongoing political and security instability since 2012, all of which strain service provision and widen existing inequities. These challenges persist despite major reform efforts, including the 2019 initiative to provide free care for children under five and adults over seventy, which has been slowed by funding gaps, the COVID-19 pandemic and the current global health funding crisis.<br><br>In this context, understanding both the geographic and facility-level barriers that shape access to care is crucial. By estimating the number of people affected by travel-time constraints and by the unavailability of ART services at nearby facilities, this study provides a more comprehensive view of access than models focused solely on physical distance. Using HIV services as a case study, we offer evidence that can support more targeted planning and help ensure that health system reforms address the most pressing barriers to service delivery in Mali.
2. How were local researchers involved in study design?	The Health Resources and Services Availability Monitoring System (HeRAMS) is a collaborative initiative between country teams and WHO headquarters designed to systematically collect and share core information on essential health resources and services. In Mali, the coordination of data collection, data ownership, and overall system management is led locally by the HeRAMS team in Bamako, in close collaboration with

	the Ministry of Health. This study is based on a secondary analysis of these routinely collected HeRAMS data. Work on the data and its contextualization for this study was conducted primarily with OT, with additional support from HC and OS.
**Research management**	
3. How has funding been used to support the local research team(s)?	This study did not require any additional grants or funding, as it relied on secondary analysis of HeRAMS data already collected as part of the ongoing program. Apart from the existing contracts of the individual researchers and the collaboration between the University of Geneva and HeRAMS—through which WHO Geneva funded the UNIGE researchers—no further financial support was needed. Colleagues at HeRAMS Bamako were supported through their existing WHO contracts.
**Data acquisition and analysis**	
4. How are research staff who conducted data collection acknowledged?	All individuals involved in this secondary data analysis, for which no new data collection was required, have been included as co-authors on the paper.
5. How have members of the research partnership been provided with access to study data?	As this study is a secondary data analysis of an existing and ongoing data collection effort on health service delivery units and their associated services and resources, data ownership remains with the country and Ministry of Health.
6. How were data used to develop analytical skills within the partnership?	The first author (PT) conducted the primary data analysis, and OT was responsible for extracting the data from the database. All scripts and modelling components have been incorporated into the wider HeRAMS analysis pipeline to support reproducibility.
**Data interpretation**	
7. How have research partners collaborated in interpreting study data?	PT and FH led the initial drafting and interpretation of the results, but all findings and any inconsistencies in the data were thoroughly discussed with OT through online meetings and email exchanges. During the writing stage, all Mali-based co-authors reviewed the text and figures and contributed additional contextual information where needed, particularly regarding the Malian

	health system.
**Drafting and revising for intellectual content**	
8. How were research partners supported to develop writing skills?	The lead author (PT) and second author (FH) have both published several papers and wrote the first full drafts in collaboration which were then reviewed by all co-authors and refined were needed.
9. How will research products be shared to address local needs?	All HeRAMS data can be requested through the HeRAMS platform, and regular updates of the baseline analysis for Mali are publicly available through the WHO website. The outputs of this study can be shared upon reasonable request, provided that local partners agree to the dissemination, as health facility coordinates may be sensitive in certain contexts. All modelling work is open source through AccessMod and can be fully reproduced.
**Authorship**	
10. How is the leadership, contribution and ownership of this work by LMIC researchers recognised within the authorship?	This is recognised through the co-authorship of three Malian based co-authors OT, HC, and OS. All their contributions have been recognized in the contributor statement in the manuscript.
11. How have early career researchers across the partnership been included within the authorship team?	The first and second author are both within the first 5 years of finishing their PhD and thus more junior researchers.
12. How has gender balance been addressed within the authorship?	Two out of the eight authors identify as women and the other as men.
**Training**	
13. How has the project contributed to training of LMIC researchers?	Our secondary analysis did not involve direct training of researchers. However, in parallel projects and capacities, we have supported capacity-building efforts in Mali over the last years. For example, in collaboration with UNFPA, we organized training sessions and a prioritization workshop on accessibility modelling for emergency obstetric care.
**Infrastructure**	
14. How has the project contributed to improvements in local infrastructure?	This project did not contribute to improvements in local infrastructure, as it was based on secondary data analysis and relied on databases and data pipelines that were already in place.

Governance	
15. What safeguarding procedures were used to protect local study participants and researchers?	Since our data reflect health service delivery units rather than individual-level information, there were no local study participants involved, and no specific ethical or safeguarding procedures were required for individuals. However, because the locations of health facilities can be sensitive and may pose security risks in some settings, we did not release the precise facility coordinates.
