# Supplementary material for: Quantifying the impact of health service delivery barriers on access to healthcare: a case study of antiretroviral therapy in Mali
Source: BMJ Glob Health. 2026 Jun 19;11(6):e019476. doi: 10.1136/bmjgh-2025-019476 (PMC13289087; doi:10.1136/bmjgh-2025-019476)
Supplement: online supplemental file 2 [file bmjgh-11-6-s002.pdf]

Supplemental material

Supplemental Table 1. Input data including their types and sources.

Dataset	Producer	Resolution	Year	Citation
**Health facilities**	HeRAMS	Vectorized	2023	[17]
**Administrative boundaries**	Geographic Institute of Mali (IGM)	Vectorized	2024	-
**Roads**	Geographic Institute of Mali (IGM)	Vectorized	2022	-
**Rivers**	Geographic Institute of Mali (IGM)	Vectorized	2022	-
**Digital elevation model**	Shuttle Radar Topography Mission (SRTM)	90 m	2013	[20]
**Land cover**	Copernicus Global Land Service	100 m	2020	[19]
**Population**	WorldPop and National Office of Reproductive Health (ONASR)	90 m	2022	[18]
**Sub-national travel scenarios**	Local experts	Table	2022	-

Supplemental Table 2. Travel scenario for three eco-geographical zones in Mali.

Scenario	Regions	Label	Mode	Speed (km/h)
Scenario 1	Kayes, Koulikoro, Ségou, Bamako, Sikasso	AMELIOREE	MOTORIZED	40
Scenario 1	Kayes, Koulikoro, Ségou, Bamako, Sikasso	BITUME	MOTORIZED	50
Scenario 1	Kayes, Koulikoro, Ségou, Bamako, Sikasso	Bac	MOTORIZED	14
Scenario 1	Kayes, Koulikoro, Ségou, Bamako, Sikasso	ECHANGEUR_VOIE_ACCES	MOTORIZED	40
Scenario 1	Kayes, Koulikoro, Ségou, Bamako, Sikasso	GROSSE_PISTE	MOTORIZED	40
Scenario 1	Kayes, Koulikoro, Ségou, Bamako, Sikasso	NR	MOTORIZED	50
Scenario 2	Mopti	bare / sparse vegetation	WALKING	3
Scenario 1	Kayes, Koulikoro, Ségou, Bamako, Sikasso	TERRE_MODERNE	MOTORIZED	40
Scenario 1	Kayes, Koulikoro, Ségou, Bamako, Sikasso	VOIE_URBAINE_BITUMEE	MOTORIZED	40
Scenario 1	Kayes, Koulikoro, Ségou, Bamako, Sikasso	VOIE_URBAINE_NON_BITUMEE	MOTORIZED	30
Scenario 1	Kayes, Koulikoro, Ségou, Bamako, Sikasso	bare / sparse vegetation	MOTORIZED	20
Scenario 3	Tombouctou, Gao, Menaka, Kidal, Taoudénit	bare / sparse vegetation	WALKING	3
Scenario 1	Kayes, Koulikoro, Ségou, Bamako, Sikasso	cropland	MOTORIZED	20
Scenario 1	Kayes, Koulikoro, Ségou, Bamako, Sikasso	closed forest unknown	WALKING	1.5
Scenario 2	Mopti	cropland	WALKING	3
Scenario 3	Tombouctou, Gao, Menaka, Kidal, Taoudénit	cropland	WALKING	3
Scenario 1	Kayes, Koulikoro, Ségou, Bamako, Sikasso	herbaceous vegetation	MOTORIZED	20
Scenario 1	Kayes, Koulikoro, Ségou, Bamako, Sikasso	deciduous broadleaf closed forest	WALKING	1.5
Scenario 1	Kayes, Koulikoro, Ségou, Bamako, Sikasso	deciduous broadleaf open forest	WALKING	1.5
Scenario 2	Mopti	deciduous broadleaf open forest	WALKING	1.5
Scenario 1	Kayes, Koulikoro, Ségou, Bamako, Sikasso	shrubs	MOTORIZED	20
Scenario 1	Kayes, Koulikoro, Ségou, Bamako, Sikasso	urban	MOTORIZED	40
Scenario 2	Mopti	AMELIOREE	MOTORIZED	45
Scenario 2	Mopti	BITUME	MOTORIZED	50
Scenario 2	Mopti	GROSSE_PISTE	MOTORIZED	40
Scenario 2	Mopti	NR	MOTORIZED	50
Scenario 3	Tombouctou, Gao, Menaka, Kidal, Taoudénit	deciduous broadleaf open forest	WALKING	1.5
Scenario 2	Mopti	TERRE_MODERNE	MOTORIZED	40
Scenario 2	Mopti	VOIE_URBAINE_BITUMEE	MOTORIZED	40
Scenario 2	Mopti	VOIE_URBAINE_NON_BITUMEE	MOTORIZED	30
Scenario 1	Kayes, Koulikoro, Ségou, Bamako, Sikasso	evergreen broadleaf closed forest	WALKING	1.5
Scenario 2	Mopti	herbaceous vegetation	WALKING	3
Scenario 3	Tombouctou, Gao, Menaka, Kidal, Taoudénit	herbaceous vegetation	WALKING	3
Scenario 1	Kayes, Koulikoro, Ségou, Bamako, Sikasso	herbaceous wetland	WALKING	1.5
Scenario 2	Mopti	herbaceous wetland	WALKING	1.5
Scenario 3	Tombouctou, Gao, Menaka, Kidal, Taoudénit	herbaceous wetland	WALKING	1.5
Scenario 2	Mopti	permanent water bodies	MOTORIZED	20
Scenario 1	Kayes, Koulikoro, Ségou, Bamako, Sikasso	open forest unknown	WALKING	1.5
Scenario 2	Mopti	urban	MOTORIZED	40
Scenario 3	Tombouctou, Gao, Menaka, Kidal, Taoudénit	BITUME	MOTORIZED	60
Scenario 3	Tombouctou, Gao, Menaka, Kidal, Taoudénit	GROSSE_PISTE	MOTORIZED	50
Scenario 3	Tombouctou, Gao, Menaka, Kidal, Taoudénit	NR	MOTORIZED	60
Scenario 2	Mopti	open forest unknown	WALKING	1.5
Scenario 3	Tombouctou, Gao, Menaka, Kidal, Taoudénit	TERRE_MODERNE	MOTORIZED	50
Scenario 3	Tombouctou, Gao, Menaka, Kidal, Taoudénit	VOIE_URBAINE_BITUMEE	MOTORIZED	40
Scenario 3	Tombouctou, Gao, Menaka, Kidal, Taoudénit	VOIE_URBAINE_NON_BITUMEE	MOTORIZED	30
Scenario 3	Tombouctou, Gao, Menaka, Kidal, Taoudénit	open forest unknown	WALKING	1.5
Scenario 1	Kayes, Koulikoro, Ségou, Bamako, Sikasso	permanent water bodies	WALKING	0
Scenario 1	Kayes, Koulikoro, Ségou, Bamako, Sikasso	PETITE_PISTE	WALKING	3
Scenario 2	Mopti	PETITE_PISTE	WALKING	3
Scenario 3	Tombouctou, Gao, Menaka, Kidal, Taoudénit	PETITE_PISTE	WALKING	3
Scenario 2	Mopti	shrubs	WALKING	1.5
Scenario 3	Tombouctou, Gao, Menaka, Kidal, Taoudénit	permanent water bodies	MOTORIZED	20
Scenario 3	Tombouctou, Gao, Menaka, Kidal, Taoudénit	shrubs	WALKING	1.5
Scenario 3	Tombouctou, Gao, Menaka, Kidal, Taoudénit	urban	MOTORIZED	40

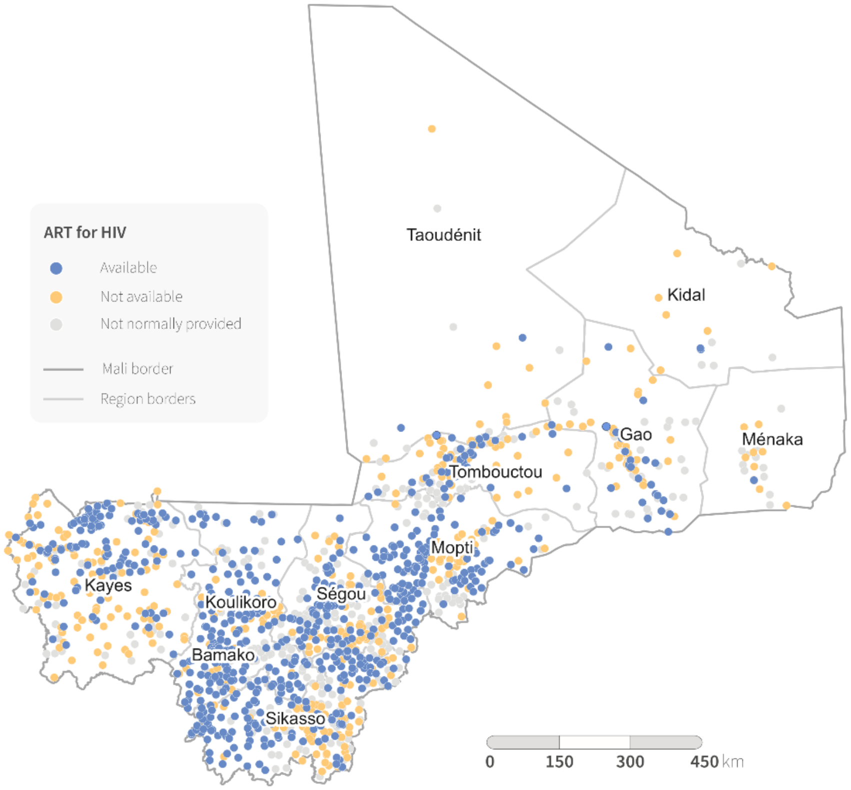

Supplemental Figure 1. Location of public health facilities in Mali. Labels refer to the ten Mali regions and the Bamako capital district. Colors inform about the availability status of the ART for HIV at the facility level.

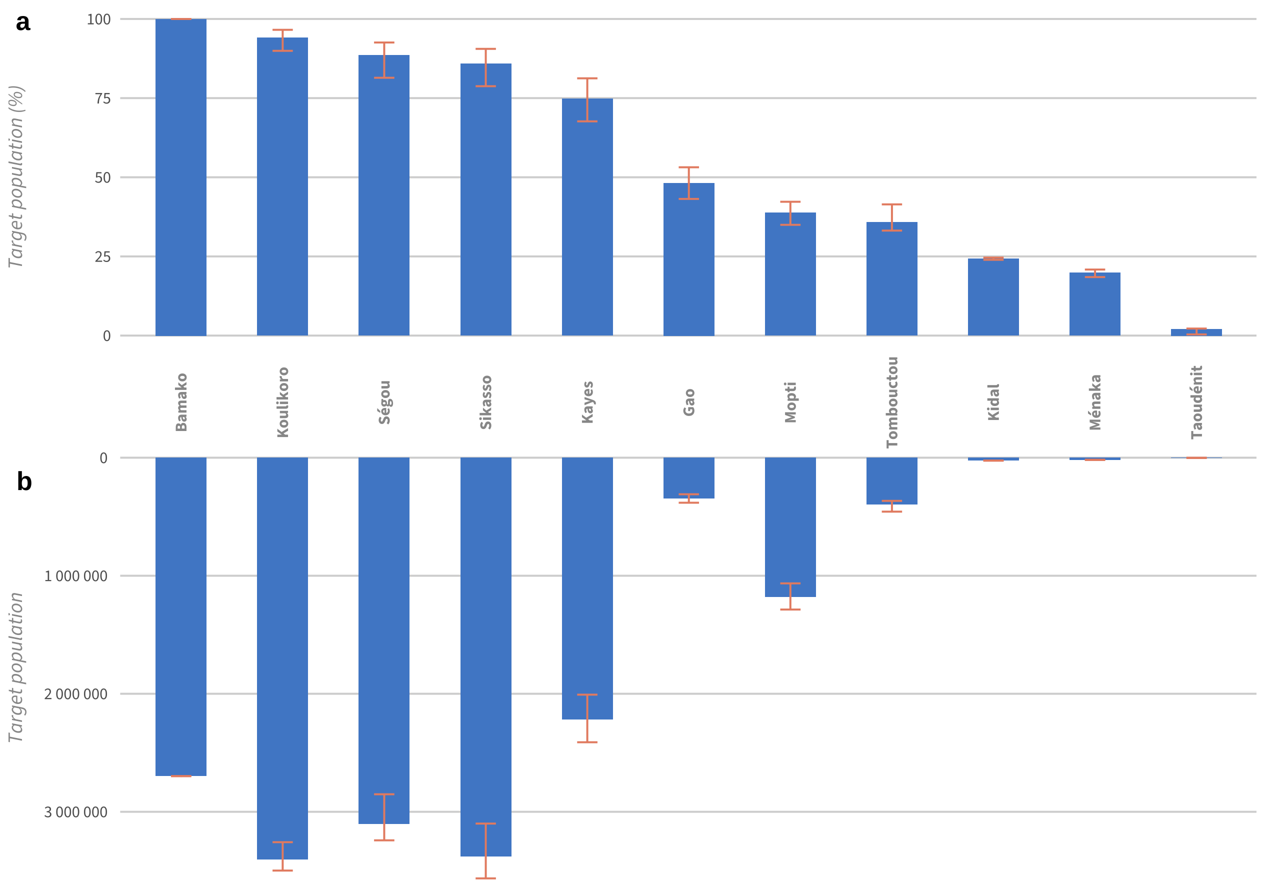

Supplemental Figure 2. *The accessibility coverage of ART within 60 minutes in a) relative and b) absolute values by region. The regions in the barplots are arranged in descending order of relative population coverage. Orange error bars indicate the coverage uncertainty, considering -20% and +20% on travel speeds.*

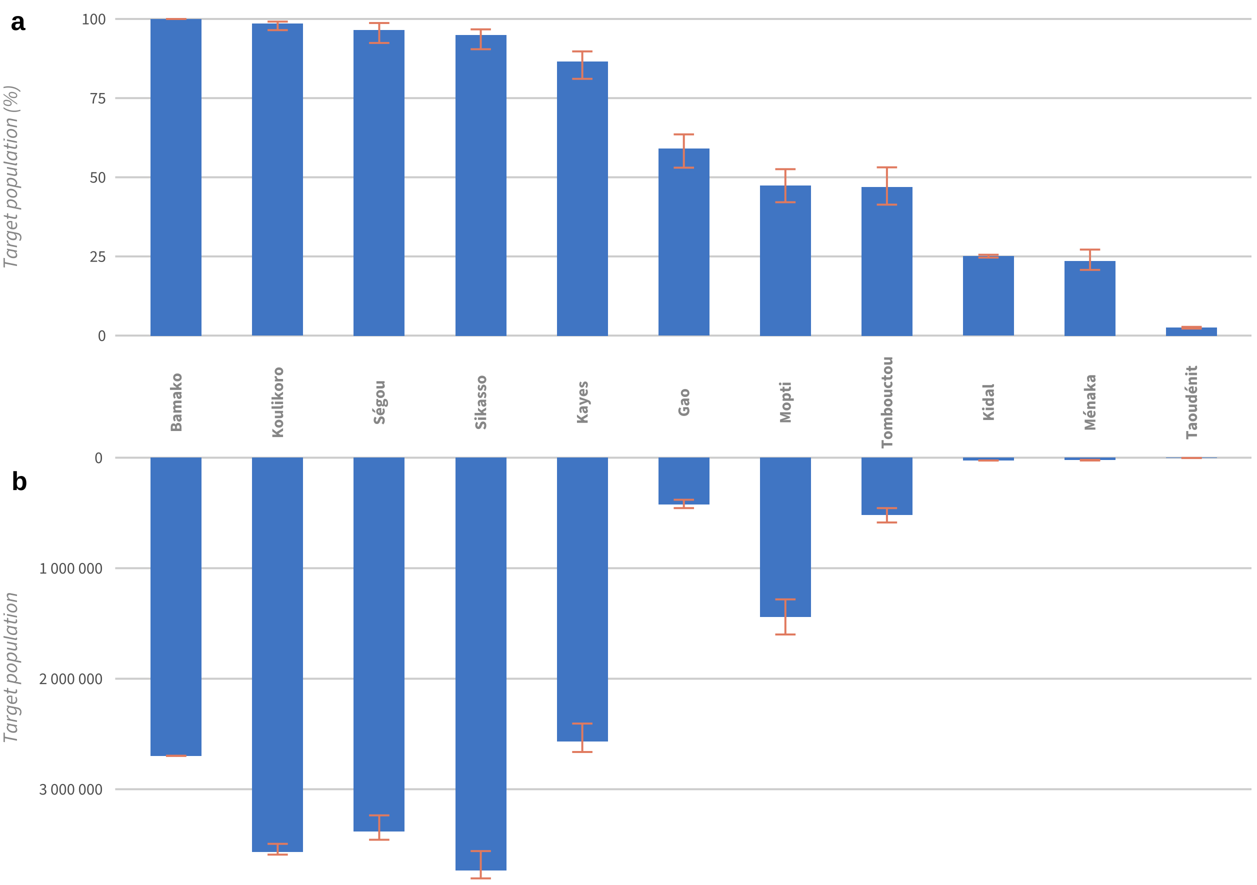

Supplemental Figure 3. *The accessibility coverage of ART within 90 minutes in a) relative and b) absolute values by region. The regions in the barplots are arranged in descending order of relative population coverage. Orange error bars indicate the coverage uncertainty, considering -20% and +20% on travel speeds.*

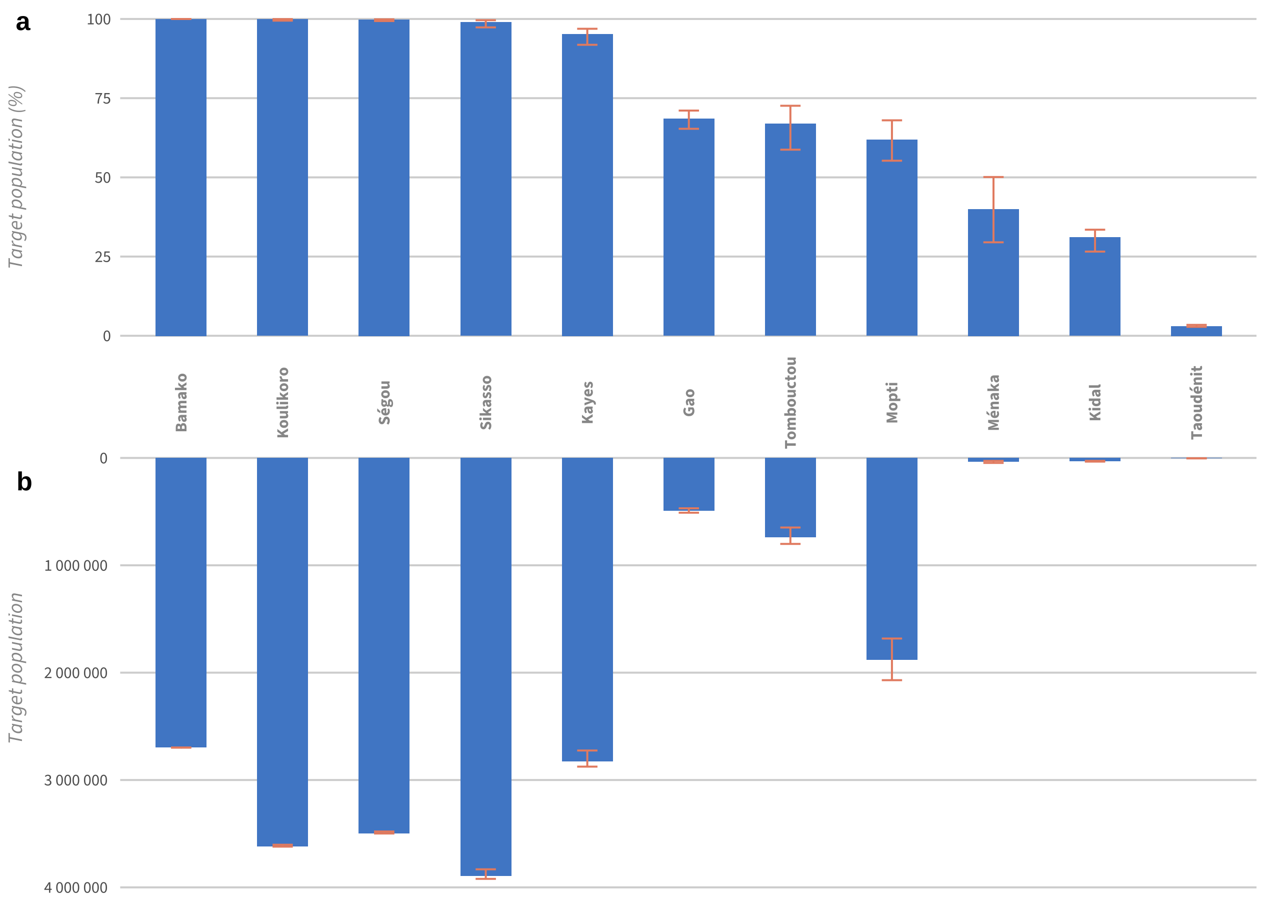

Supplemental Figure 4. *The accessibility coverage of ART within 150 minutes in a) relative and b) absolute values by region. The regions in the barplots are arranged in descending order of relative population coverage. Orange error bars indicate the coverage uncertainty, considering -20% and +20% on travel speeds.*

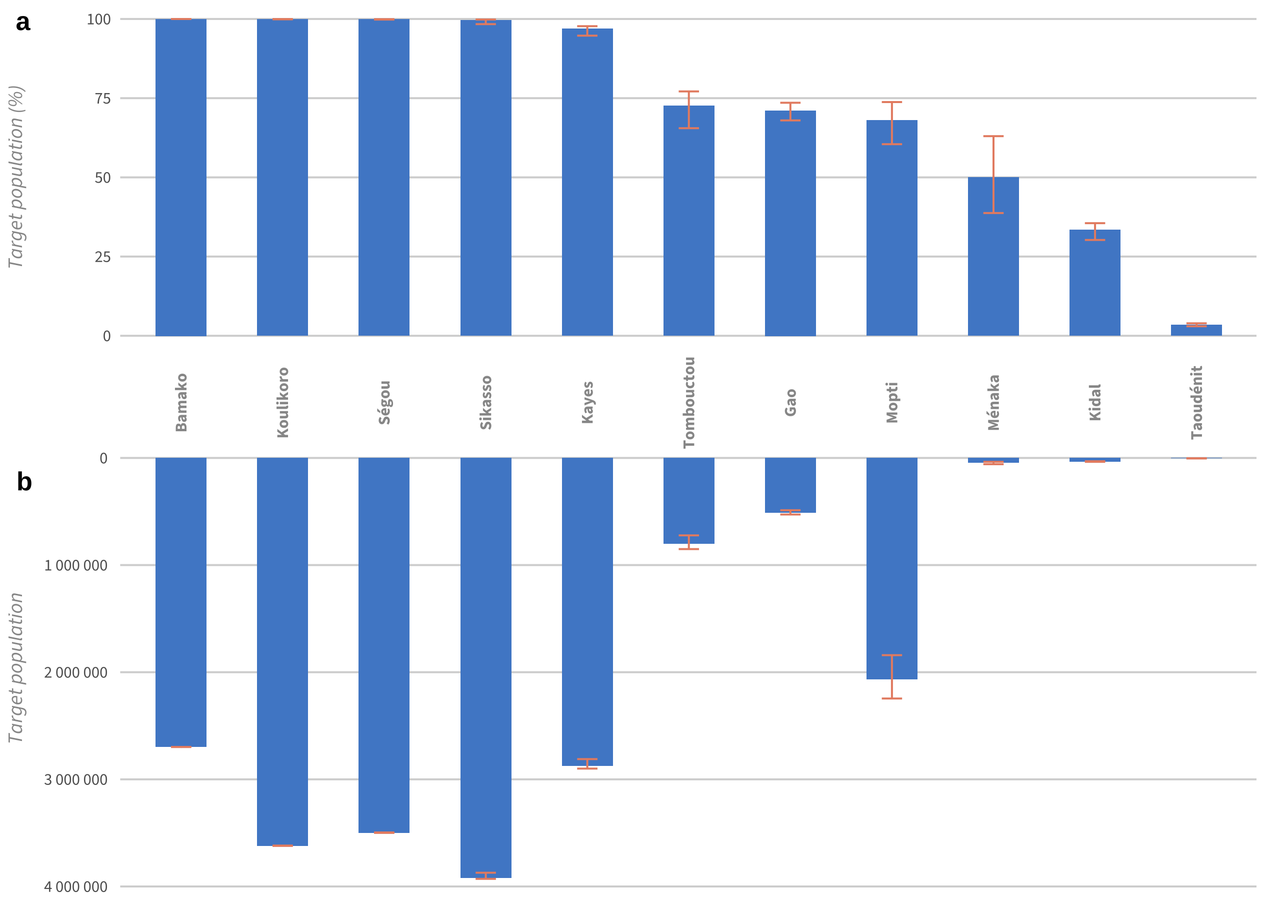

Supplemental Figure 5. *The accessibility coverage of ART within 180 minutes in a) relative and b) absolute values by region. The regions in the barplots are arranged in descending order of relative population coverage. Orange error bars indicate the coverage uncertainty, considering -20% and +20% on travel speeds.*

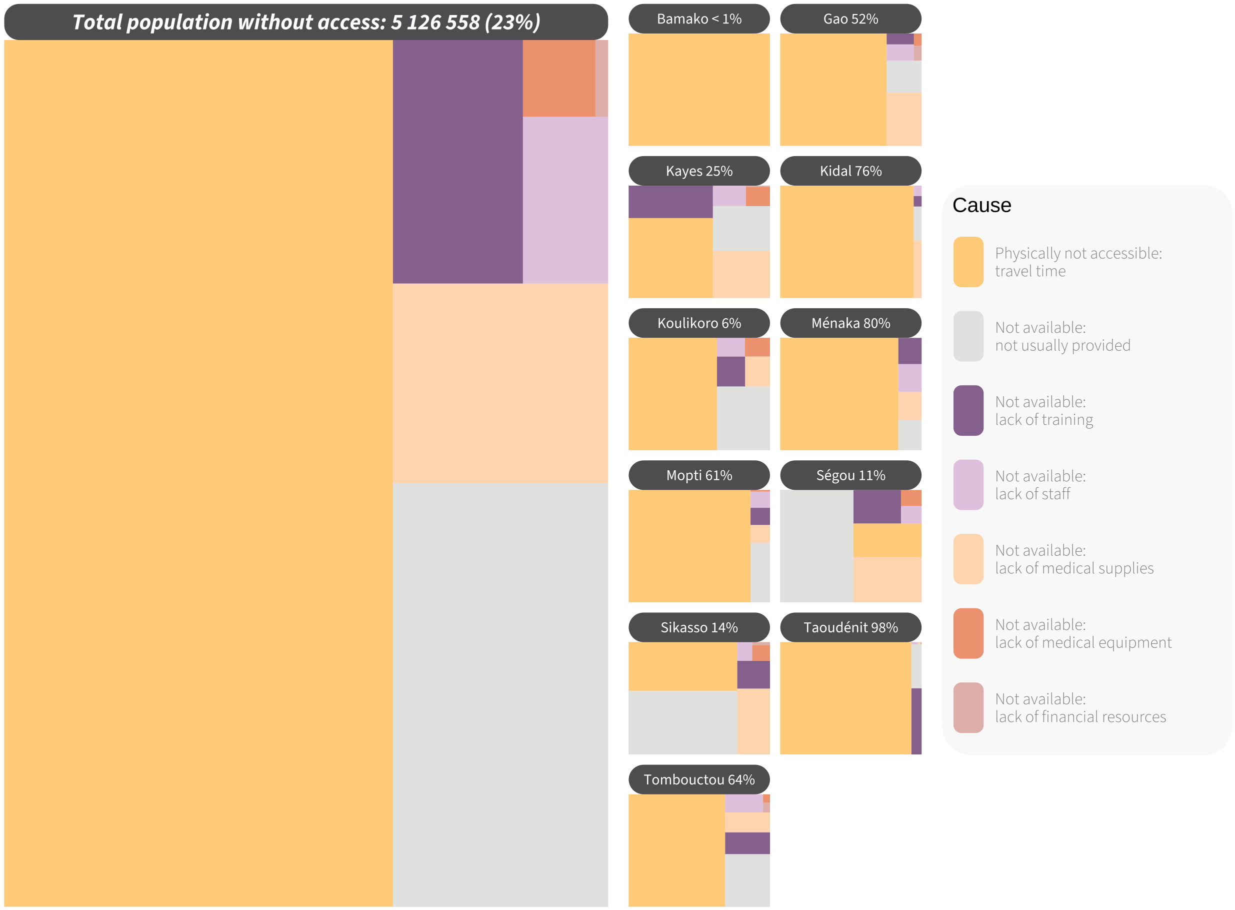

Supplemental Figure 6. Treemap representing the causes of inaccessibility to the antiretroviral therapy (ART) in Mali for a maximum travel time of 60 minutes, according to the number of affected people. The percentages indicated for each region represent the percentage of the population without access to the service. The relative size of each cause of inaccessibility within a region is proportional to the percentage of the affected population.

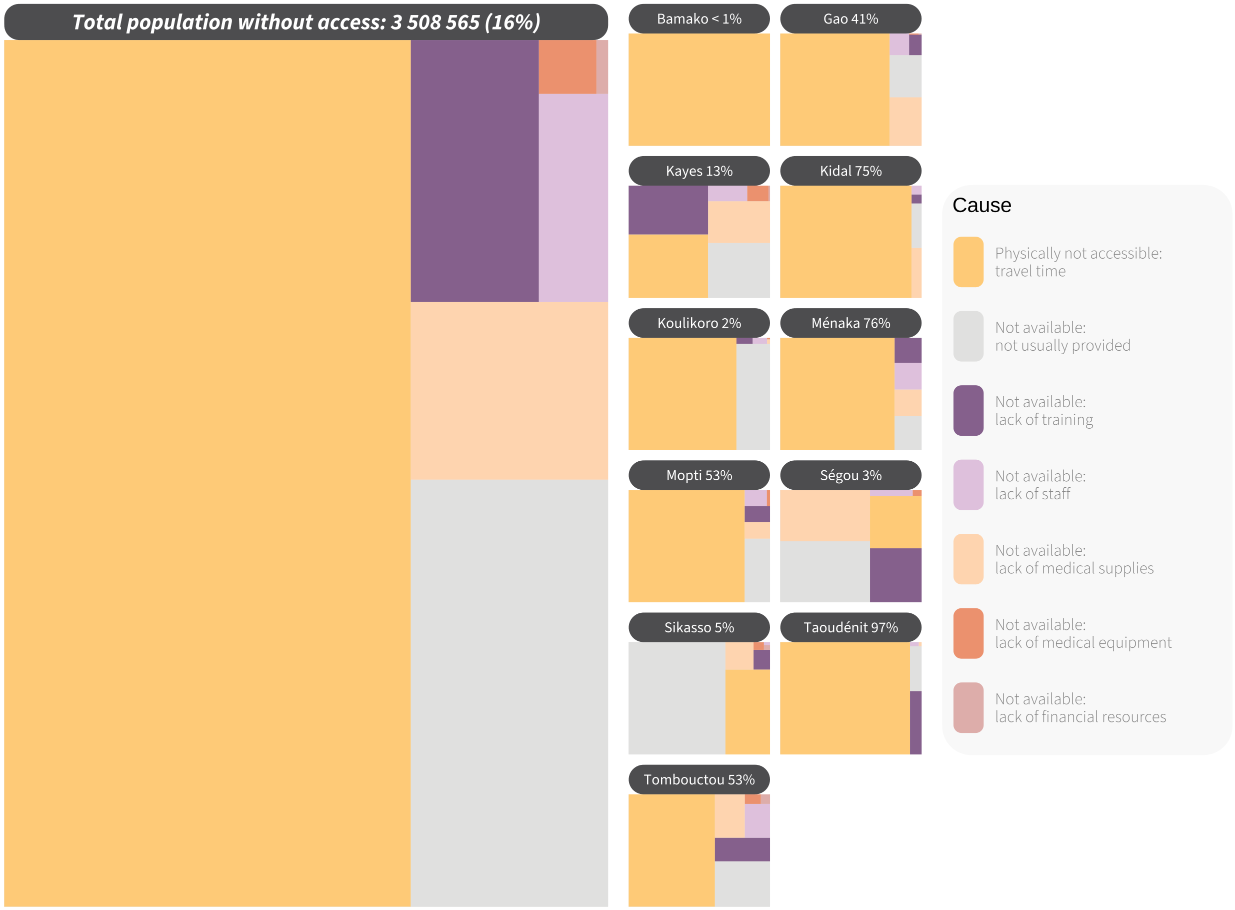

Supplemental Figure 7. Treemap representing the causes of inaccessibility to the antiretroviral therapy (ART) in Mali for a maximum travel time of 90 minutes, according to the number of affected people. The percentages indicated for each region represent the percentage of the population without access to the service. The relative size of each cause of inaccessibility within a region is proportional to the percentage of the affected population.

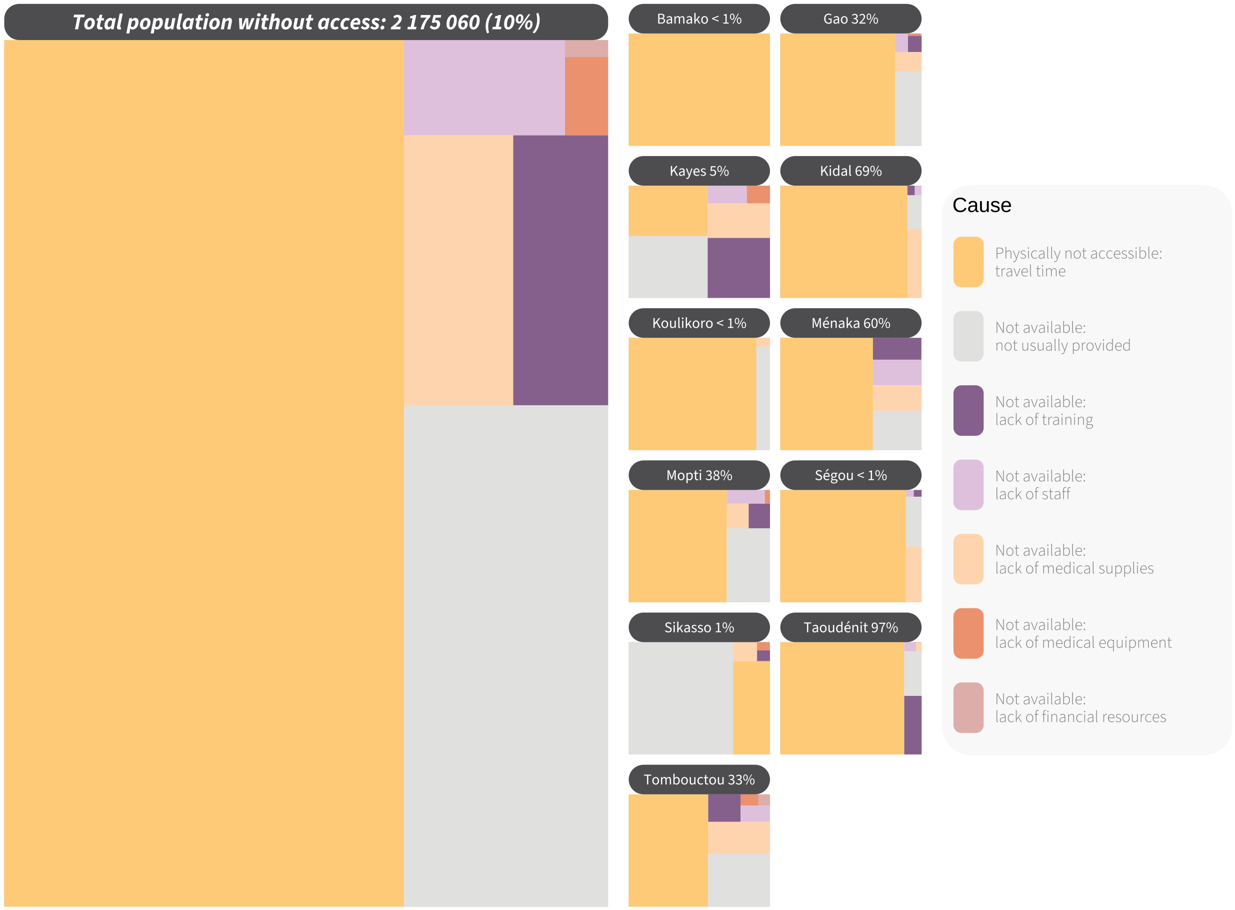

Supplemental Figure 8. Treemap representing the causes of inaccessibility to the antiretroviral therapy (ART) in Mali for a maximum travel time of 150 minutes, according to the number of affected people. The percentages indicated for each region represent the percentage of the population without access to the service. The relative size of each cause of inaccessibility within a region is proportional to the percentage of the affected population.

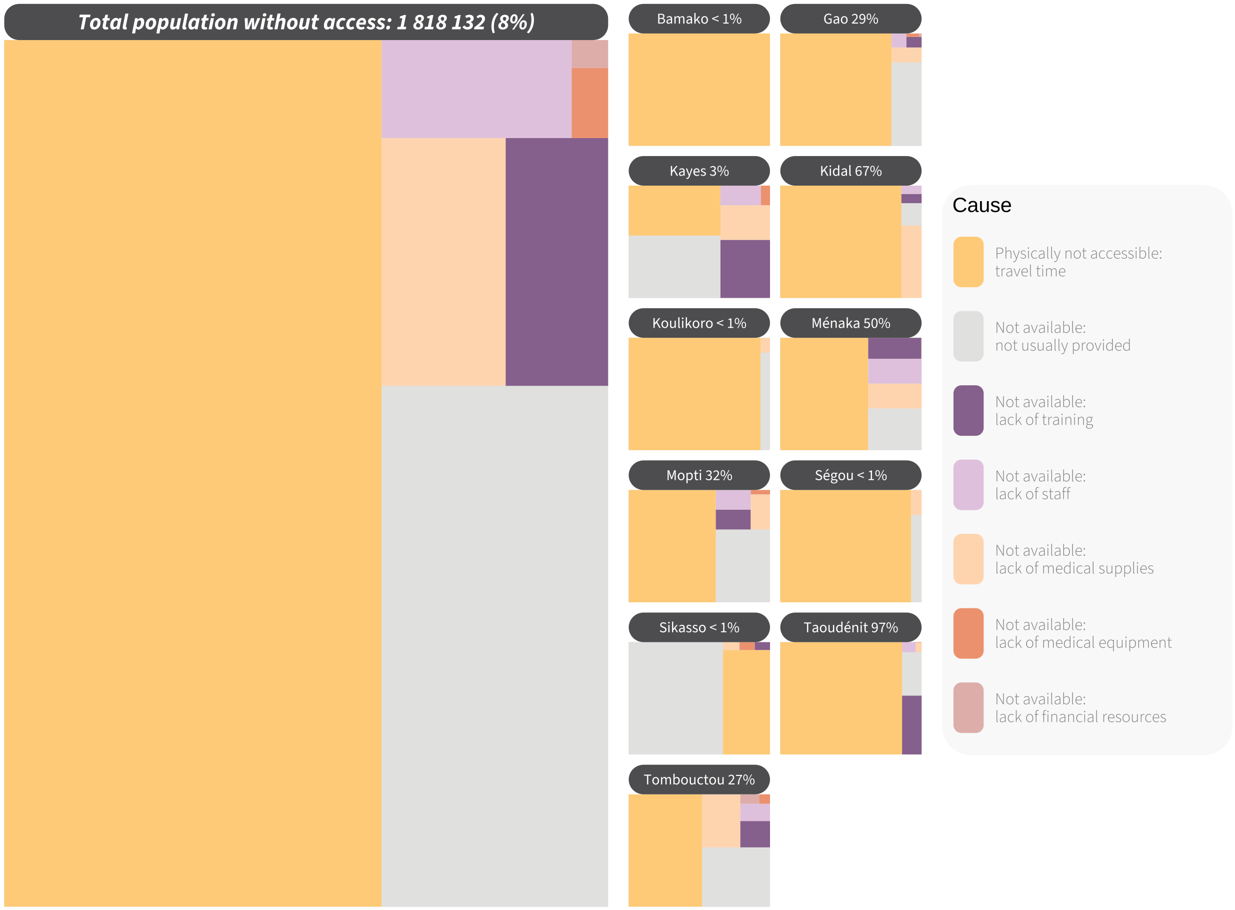

Supplemental Figure 9. Treemap representing the causes of inaccessibility to the antiretroviral therapy (ART) in Mali for a maximum travel time of 180 minutes, according to the number of affected people. The percentages indicated for each region represent the percentage of the population without access to the service. The relative size of each cause of inaccessibility within a region is proportional to the percentage of the affected population.

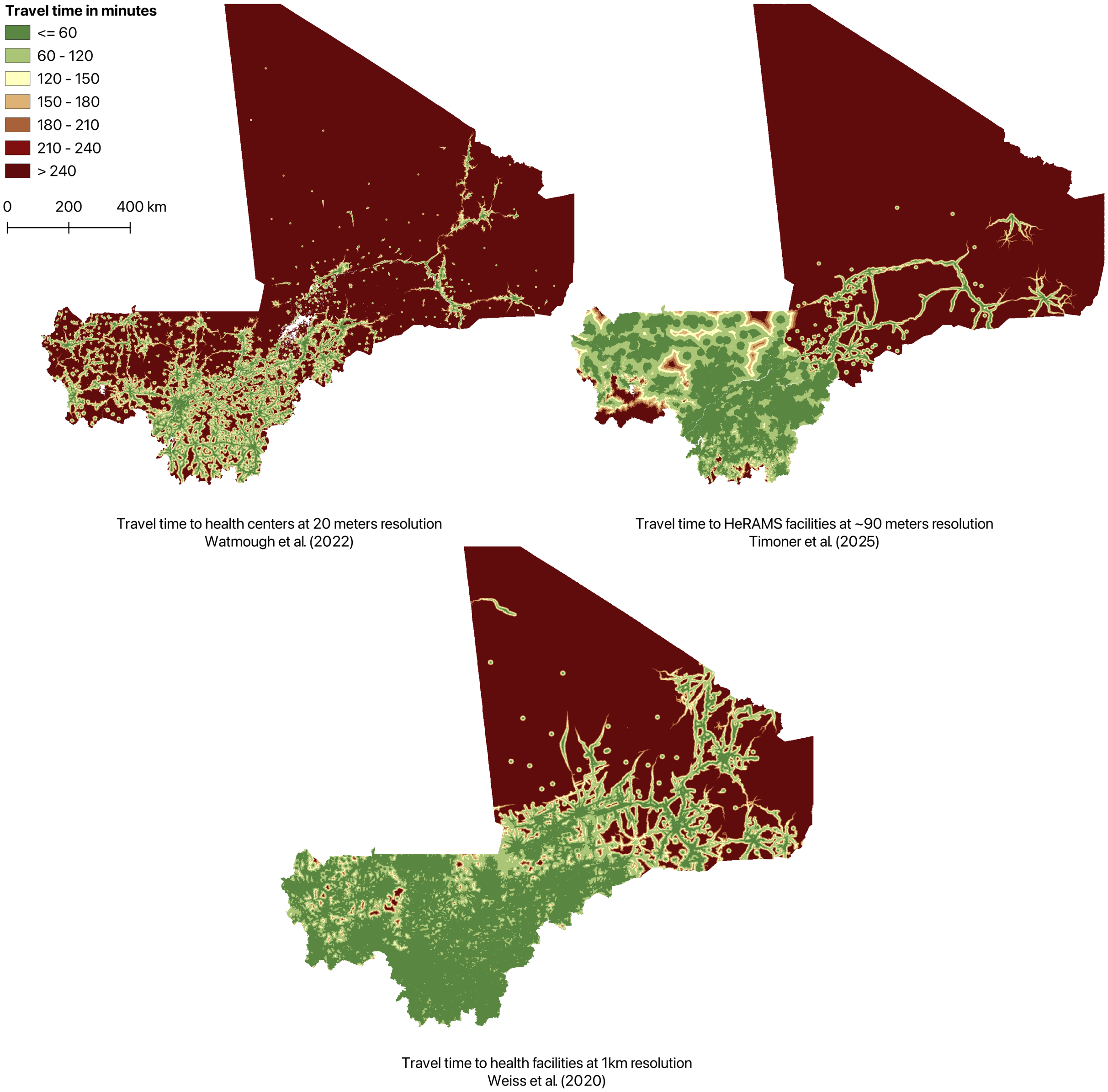

Supplemental Figure 10. Comparative maps of travel time to health facilities in Mali from three studies: (1) travel time calculated at 20 m resolution by Watmough et al. (2022); (2) travel time calculated in this study by Timoner et al. (2025); and (3) travel time calculated at 1 km resolution by Weiss et al. (2020).
